# Ultrasound-Assisted Encapsulation of Phytochemicals for Food Applications: A Review

**DOI:** 10.3390/foods12203859

**Published:** 2023-10-21

**Authors:** Vitoria Hagemann Cauduro, Jiwei Cui, Erico Marlon Moraes Flores, Muthupandian Ashokkumar

**Affiliations:** 1 Department of Chemistry, Federal University of Santa Maria, Santa Maria 97105-900, RS, Brazil; vitoriahcauduro@gmail.com (V.H.C.); ericommf@gmail.com (E.M.M.F.); 2 School of Chemistry and Chemical Engineering, Shandong University, Jinan 250100, China; jwcui@sdu.edu.cn; 3 School of Chemistry, The University of Melbourne, Parkville, VIC 3010, Australia

**Keywords:** encapsulation, ultrasound, phytochemicals, bioactive compounds, functional food, food technology, process intensification

## Abstract

The use of phytochemicals as natural food additives is a topic of interest for both academic and food industry communities. However, many of these substances are sensitive to environmental conditions. For this reason, encapsulation is usually performed prior to incorporation into food products. In this sense, ultrasound-assisted encapsulation is an emerging technique that has been gaining attention in this field, bringing important advantages for the production of functional food products. This review article covered applications published in the last five years (from 2019 to 2023) on the use of ultrasound to encapsulate phytochemicals for further incorporation into food. The ultrasound mechanisms for encapsulation, its parameters, such as reactor configuration, frequency, and power, and the use of ultrasound technology, along with conventional encapsulation techniques, were all discussed. Additionally, the main challenges of existing methods and future possibilities were discussed. In general, ultrasound-assisted encapsulation has been considered a great tool for the production of smaller capsules with a lower polydispersity index. Encapsulated materials also present a higher bioavailability. However, there is still room for further developments regarding process scale-up for industrial applications. Future studies should also focus on incorporating produced capsules in model food products to further assess their stability and sensory properties.

## 1. Introduction

Over the last decades, phytochemicals have been regarded with increasing interest due to their bioactive properties. With the incorporation of these compounds in food, it is possible to obtain functionalized food products with increased nutritional/health benefits [1]. A great advantage of these phytochemicals is that, as these compounds can be obtained from plants, they can be extracted from agricultural by-products to obtain value-added substances that can replace synthetic formulations as additives in food products [2]. In recent years, a big effort has been made for the extraction, isolation, analysis, and application of phytochemicals in the food industry [3,4]. However, many phytochemical compounds cannot be directly incorporated into food products or even packaging materials due to their chemical instability, poor solubility, or unfavorable effects on the food quality (e.g., flavor, appearance, texture, etc.). 

However, some problems can occur when incorporating phytochemicals into food products due to compatibility issues with the matrix or sensibility to external conditions, which can lead to degradation and loss of functionality [5]. In order to increase the shelf life of these compounds and protect against degradation, the introduction of a physical layer between the phytochemical and the food matrix brings many advantages, such as improved chemical stability, water dispersibility, and compatibility with the food medium [6,7,8,9,10,11]. In this sense, many encapsulation methodologies have been developed, and some well-established techniques are available, such as spray drying [12] and emulsification using high-speed homogenizers [13]. 

Process intensification using ultrasound (US) is also a fast growing field, as it enables higher efficiency, lower reaction times, and the use of milder reaction conditions [14]. Ultrasound applications have presented continuous growth, being extensively used in food processing for drying, crystallization, extraction, microbial inactivation, emulsification, and other processes [15,16]. Specifically, low-frequency US has been increasingly explored for emulsification and nutrient encapsulation [17,18]. In this sense, this energy source can be considered a good alternative to conventional techniques for phytochemical encapsulation, being used as a stand-alone method or in conjunction with other methodologies [19].

For this review article, a search on the Web of Science database (Clarivate Analytics) was carried out. The keywords “ultrasound”, “encapsulation”, and “phytochemical” were used. Specific classes of phytochemicals were also used as keywords (i.e., alkaloids, phytosterols, polyphenols, terpenoids, or organosulfur compounds). Studies in which US was used in the capsule production step were considered. In this review, the use of US technology, along with different encapsulation techniques for phytochemical encapsulation, was discussed, covering the period from January of 2019 to September of 2023. The US experimental parameters, as well as the reactor configurations used for these methods, were also covered. In addition, the most used core and shell materials, as well as the incorporation of produced capsules into real food products and the possibility of scaling up, were discussed.

As it will be discussed in this review, it was observed that these applications were mainly focused on the encapsulation of polyphenols. Furthermore, emulsification has been the preferred encapsulation technique using US technology. However, there was a general lack of information regarding important parameters related to the use of US systems, such as frequency, power, and amplitude, which makes it hard to reproduce the reported methods. Finally, there was still room for further developments in this field regarding US parameter optimizations and the incorporation of produced capsules into food models for sensory and stability testing. 

## 2. Phytochemicals in Functional Food Products

Phytochemicals, or phytonutrients, are secondary metabolites produced by plants with the objective of protecting them from external stress, such as ultraviolet radiation, bacteria, or pollutants [20]. Thousands of these substances exist, but relatively few have been effectively isolated from plant materials [1]. These phytochemicals are classified, according to their structure and functionality, as polyphenols, alkaloids, phytosterols, terpenoids, or organosulfur compounds [21]. Polysaccharides have also been classified as phytochemicals, though not as commonly [22]. Out of these classes, the class of polyphenols has been the most studied due to its unique properties, such as its antioxidant, anti-inflammatory, antimicrobial, and anticancer activities, as well as its cardioprotective effects [23]. The structures of polyphenols can vary from small molecules, such as gallic acid, to complex macromolecular compounds, such as oligomeric proanthocyanidins. This class is characterized by the presence of aromatic rings containing one or more hydroxyl substituents, with flavonoids being its most common sub-class [2,8]. An example of these compounds is quercetin, a common flavonoid found in several plants, such as onions and broccoli, which is known to exhibit anti-inflammatory and anticancer activity [20]. Another common example is curcumin, which is a polyphenol that has been reported to have many beneficial effects, such as anti-neurodegenerative and anti-diabetes activity [20]. Hence, including these compounds in food products that may not naturally have these properties (such as dairy products, which are widely consumed worldwide) can result in an overall enhancement of the food’s health benefits.

In order to incorporate phytochemicals into food products, an extraction procedure must first be performed, and numerous studies have focused on developing adequate extraction methods [2,8,24,25], which will not be addressed in the present study. It is worth mentioning, however, that several studies have reported the use of crude extracts for phytochemical incorporation into food without the need for prior isolation and/or purification [8,26,27]. Nevertheless, although phytochemical compounds may have several interesting properties that can be used to enhance food properties, incorporating these substances into the food product in their pure form or as an extract might be hard. This can be due to incompatibility with the food matrix (poor incorporation of the phytochemical into the food due to certain issues, such as it being insoluble in the matrix, e.g., a hydrophobic phytochemical in a hydrophilic product), low stability, or poor sensory properties [28]. For example, the stability of phenolic compounds can be significantly affected via certain parameters, such as pH and temperature [29]. Furthermore, although the bioactivity of polyphenols is high, the bioavailability of these compounds tends to be low [30]. On the other hand, some extracts containing phytochemicals might lead to undesirable flavors or textures when incorporated into food products [28,31]. For this reason, the direct use of pure phytochemicals or plant extracts may not be feasible. In this sense, encapsulation is a promising technique to resolve these issues, as a protective layer is introduced between the substance of interest and the food matrix.

## 3. Encapsulation Techniques for Food Applications

**Figure 1:**
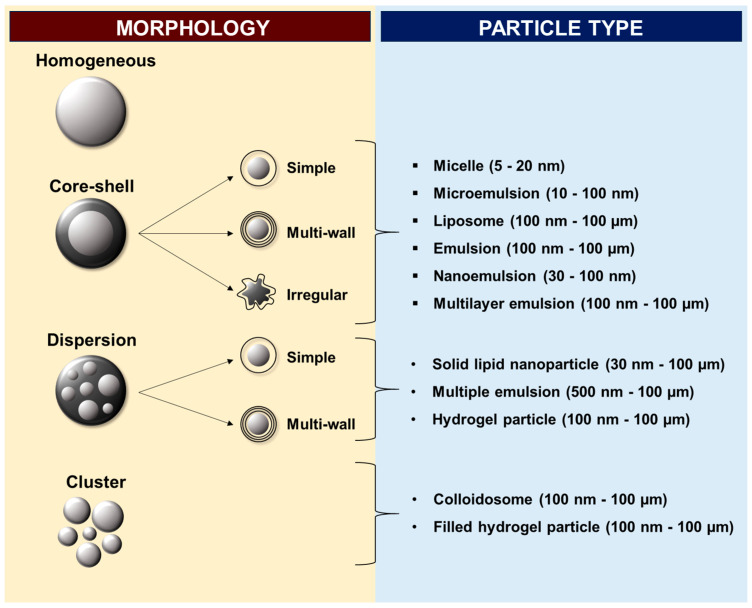
Examples of capsule morphologies that can be obtained through different encapsulation techniques, and particle types that can be produced with these morphologies. Adapted from the studies published by the authors of [28,34,35].

Encapsulation can be defined as the method through which a substance is confined in a matrix, resulting in a system with a “core” and a “shell” (also known as an encapsulant) [8,28]. These capsules can range from a few nm (nanoencapsulation) to several hundred µm (microencapsulation) in size [32,33]. Furthermore, capsule morphologies can vary greatly depending on the core and shell materials, as well as on the encapsulation technique employed (Figure 1) [28,34]. Other important characteristics, such as size distribution, stability, and crystallinity, are also dependent on these variables [33]. 

**Figure 2:**
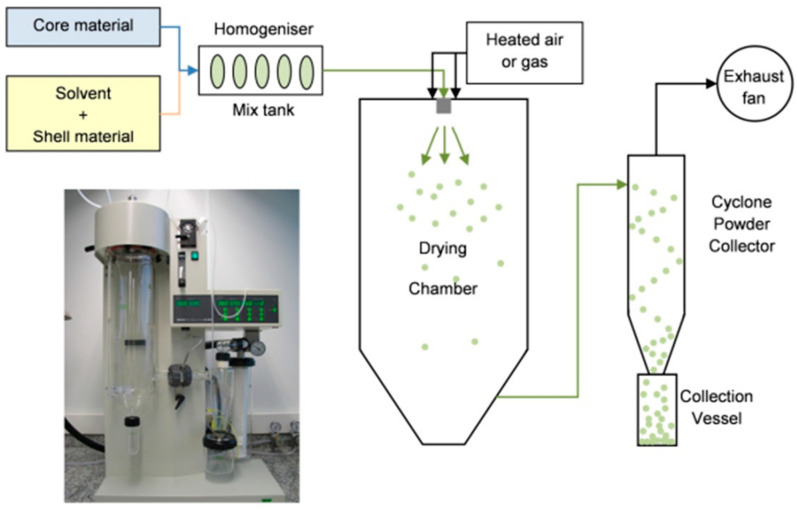
Picture of a spray dry system and a schematic representation of the spray dry encapsulation process. The liquid core and shell materials are homogenized and introduced into a drying chamber, where the droplets undergo atomization and drying to form solid capsules. Reproduced from the study published by the authors of [41].

Over the years, several encapsulation techniques have been developed with the aim of improving food functionality and increasing phytochemical stability. The choice of the most adequate encapsulation technique will depend on several factors, such as the overall cost of the encapsulation system, the production demand (i.e., the need for scaling up), the compatibility with the food matrix, the targetability for core release in the organism, the desired shelf life, and the ease of production, among others [28]. Several well-established encapsulation techniques include spray drying, emulsification [36,37,38], liposome entrapment, coacervation [39], and freeze drying [40], which are briefly described below:

- Spray drying: This technique is one of the most widely used encapsulation systems for food applications. It has been used for over 60 years, being mostly applied for flavor encapsulation [35]. For spray drying applications, the core and wall material mixture (an aqueous solution for hydrophilic core materials, or an oil-in-water emulsion for hydrophobic core materials) is passed through a nozzle into a container in which hot air is circulated (atomization), causing droplets to disperse and dry evenly, resulting in solid particles with sizes ranging from 10 to 1000 µm [28,32]. A schematic representation of this process is displayed in Figure 2.
- Emulsification: This is a popular technique for the incorporation of hydrophobic phytochemicals into food products. Emulsification consists of the mixture of immiscible liquids through shear forces, resulting in capsules with a core–shell configuration. The liquid that becomes entrapped is referred to as the dispersive phase, while the liquid present in higher volume is defined as the continuous or bulk phase. When the dispersive phase is hydrophobic, an oil-in-water emulsion is formed, and when it is hydrophilic, a water-in-oil emulsion is obtained. However, emulsions usually provide low protection against the diffusion of the encapsulated substances due to the thin shell. Hence, several strategies have been used to increase capsule stability, such as double emulsification (e.g., water-in-oil-in-water emulsions, which have a dispersion–shell capsule configuration) and lyophilization (or other drying processes) of the prepared emulsion [8,32,35].
- Liposome entrapment: This technique uses liposomes (usually phospholipids) to encapsulate materials in a core–shell configuration [8,35]. Phospholipids usually form bilayer structures with a hydrophobic inner layer, formed by the phospholipid tails, and hydrophilic outer layers, formed by the polar heads. This results in a hydrophilic core, which is compatible with hydrophilic compounds, and a hydrophobic portion in between the core and the outer layer, which is compatible with both hydrophobic and amphiphilic substances [9]. Liposomes have the advantage of being resistant to stomach digestion, being able to carry phytochemicals that are more beneficial when released in the intestines [8,35].
- Coacervation: In coacervation, the capsules are usually formed via electrostatic forces. This technique is based on liquid–liquid separation into two phases: one that is polymer-rich (coacervate) and one that is polymer-poor. The coacervate is then deposited onto the core material, coating it and generating capsules with sizes that can range from 100 to 10,000 nm. Coacervation can be defined as simple (also known as ionic gelation), in which only one polymer is used, or complex, in which two or more types of polymers are used. It is also important to mention that this technique is highly sensitive towards specific parameters, such as pH and temperature [8,28,32,35].
- Freeze drying: Also known as lyophilization or cryodesiccation, this technique is based on the use of low temperatures and vacuums to freeze and sublimate solvent molecules from a solution containing the core and wall materials, generating solid capsules with an irregular shape. Although freeze drying is considered a time-consuming technique, it can be advantageous when dealing with heat-sensitive core materials [8,32].

## 4. Ultrasound Technology in Phytochemical Encapsulation

Ultrasound can be defined as acoustic waves emitted in the range from 20 to 10,000 kHz. This range is subdivided into conventional (from 20 to 2000 kHz) and diagnostic (from 5000 to 10,000 kHz) US, with conventional US being used in the vast majority of applications regarding food processing [42]. Additionally, US can be classified in terms of intensity, which is the amount of energy carried by the wave per unit of area in a set time, generally expressed as W cm^−2^ [42]. In this sense, a division can be made, in which high-intensity US (or “power US”) is characterized by intensities higher than 1 W cm^−2^ and frequencies in the range from 20 to 100 kHz, while low-intensity US applications involve intensities lower than 1 W cm^−2^ and frequencies higher than 100 kHz (with the latter being less commonly used) [15,43]. High-intensity US has been used for several processes in food technology, such as in the extraction of bioactive compounds, emulsification, bacterial inactivation, and encapsulation [18]. In this section, the main US effects associated with encapsulation have been discussed. The different instrumental parameters, as well as the most used encapsulation mechanisms and core and shell materials, were also covered.

### 4.1. Ultrasonic Effects in a Liquid Medium

**Figure 3:**
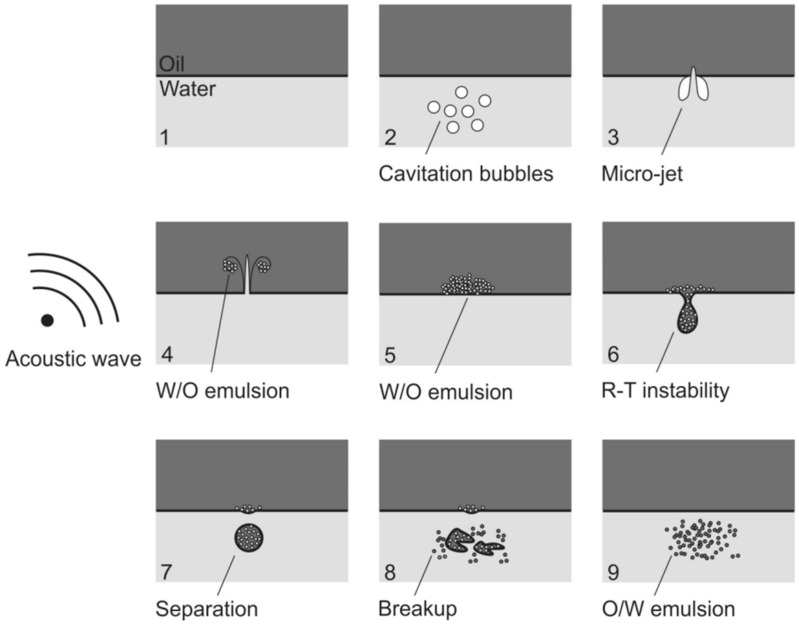
Proposed mechanism for ultrasound-assisted oil-in-water emulsification. (**1**–**5**) The collapse of cavitation bubbles causes water droplets to disperse into the oil phase, forming a water-in-oil emulsion. (**6**–**9**) Due to Rayleigh–Taylor instability, some of the oil phase disperses into the aqueous phase, and the droplets are broken down to smaller particles through continued sonication. These steps are repeated until a true oil-in-water emulsion is formed. W/O: water-in-oil; R-T: Rayleigh–Taylor; O/W: oil-in-water. Reproduced from the study published by the authors of [48].

High-intensity US, when applied to a liquid medium, results in a high mass and energy transfer, as well as high shear forces, especially in low frequencies, which are useful for food processes that are favored by turbulence in the medium, such as emulsification (e.g., the incorporation of oils into dairy products) [18]. These are a result of several physical and chemical effects, such as cavitation and acoustic streaming [44]. Acoustic cavitation is one of the major effects observed when high-intensity ultrasound systems are applied to liquid medium [45]. This effect is caused by the sequential expansion and contraction of pre-existing gases in the medium, along with the rarefaction and compression cycles of ultrasonic waves, generating bubbles or cavities [18]. These bubbles tend to grow through rectified diffusion until reaching a critical size, at which point they implode, generating shockwaves of up to 100 m s^−1^, as well as high temperatures and pressures on the microscale (~5000 K and 500 atm, respectively), which are more intense under lower US frequencies [46,47]. If bubble collapse occurs in an interfacial region, asymmetric implosion can lead to the formation of high speed, pressurized micro-jets, which can penetrate into the interface [47]. This effect, in particular, is of great relevance for emulsification processes (Figure 3). 

It is important to mention that, with continuous sonication, the temperature of the bulk solution can increase significantly, which could result in the degradation of some substances. Therefore, temperature control is essential when using ultrasound-assisted processes for food processing. Furthermore, the temperature achieved during bubble collapse can generate radical species via the homolysis of water molecules, which is more pronounced under intermediate US frequencies (from 200 to 600 kHz) [47]. 

Radical formation could be either beneficial or detrimental to food processing. On the one hand, it could potentially increase the antioxidant properties of the product via hydroxylation reactions. For example, phenol (no antioxidant activity) can be hydroxylated to form compounds such as trihydroxy phenols (antioxidants) [18]. On the other hand, sonication at high energy densities can induce the formation of a considerable amount of free radicals. This can be detrimental for food processing, causing problems, such as the oxidation of milk components and the degradation of proteins in general [17,18,43]. For this reason, process parameters should be carefully optimized in order to guarantee no unwanted effects are being caused.

A secondary, but relevant, effect of sonication is acoustic streaming, which is the swivel movement observed in a fluid when US waves are heterogeneously propagated [15,49]. A microstreaming effect can also occur, where streaming is originated from the movement of cavitation bubbles [49]. This effect is more pronounced with higher US intensities and can enhance mass transfer and increase medium temperatures. This can be useful for certain processes, such as emulsification, crystallization, drying, and others [15].

### 4.2. Specific Aspects of the Ultrasound-Assisted Encapsulation of Phytochemical Compounds

The use of US technology for the encapsulation of phytochemicals has been steadily increasing over the last five years. This energy source can be used to increase shelf life and the efficiency of conventional encapsulation techniques, being particularly useful to obtain capsules with a smaller size and size distribution [50,51]. Some key aspects of the applications published in the last five years regarding the topic of the ultrasound-assisted encapsulation of phytochemicals are shown in Table 1. 

#### 4.2.1. Ultrasonic Parameters

Sonication effects are greatly affected by the medium’s characteristics and process parameters, such as temperature, viscosity, surface tension, power, frequency, amplitude, and others [60,61]. In this sense, a careful evaluation of experimental parameters should be performed to guarantee maximum process efficiency. This is especially important, as changing some key parameters can modify the mechanisms in the reaction medium and completely change the results. A classical example is how, by only changing the frequency, the observed effects can change from emulsification (from 20 to 100 kHz) to emulsion separation (from 1000 to 3000 kHz) [62]. However, very few studies presented complete information on the applied process parameters. Furthermore, the key parameters used to understand the amount of energy effectively transferred to the reaction, i.e., acoustic intensity and density, are generally not evaluated [62]. Other extremely relevant evaluations are the use of a silent condition (or control), in order to assess the actual effects of US, and the comparison of results with conventional methods. With this in mind, there is a need for more comprehensive studies regarding US parameters’ optimization.

#### 4.2.2. Reactor Configurations

**Figure 4:**
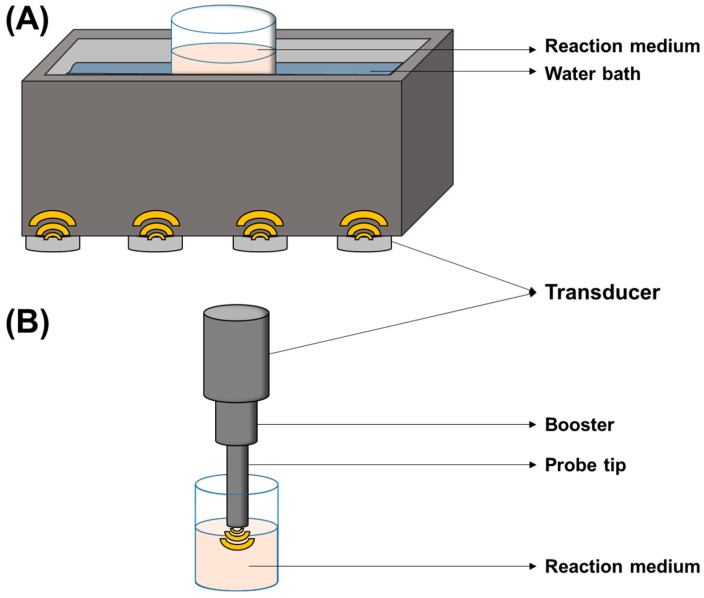
Representation of (**A**) bath and (**B**) probe reactor configurations for ultrasound-assisted encapsulation applications. In bath reactors, US is indirectly applied to the reaction medium, being transmitted from the transducers through a water bath. For probe reactors, US energy is directly transmitted into the medium via the probe tip.

The majority of applications in the last five years for phytochemical encapsulation using US have applied probe-type systems, with bath-type systems also being used in some cases. A schematic representation of these system types is shown in Figure 4. Probe-type systems consist of a transducer connected to a probe made of a titanium alloy, and propagation of the waves occurs from the tip of the probe directly into the sample. This type of application can produce higher acoustic intensities in comparison to indirect systems, such as bath sonicators, consequently generating higher shear forces, which is beneficial for obtaining smaller capsule sizes and size distribution. However, its intensity decreases exponentially with the distance from the probe tip, which makes scaling up difficult. Furthermore, these systems can quickly increase their medium temperature depending on the intensity and sonication time, which can result in undesired effects, such as protein denaturation [15,17]. For this reason, temperature control is often necessary in probe systems [42,61].

On the other hand, bath-type systems transfer US energy to the sample in an indirect way, with the waves passing through a water bath prior to reaching the reaction medium. Its main advantages are accessibility (low cost and relatively easy to obtain) and higher flexibility regarding sample volume. However, lower intensities are observed in comparison to probe-type US systems, and heating can also occur depending on the applied intensity and duration of treatment [42,61].

#### 4.2.3. Core and Shell Materials

**Figure 5:**
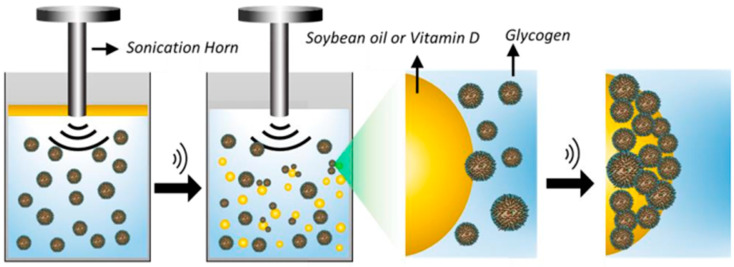
A schematic illustration of a glycogen-based shell being formed during the ultrasound-assisted encapsulation of soybean oil/vitamin D. First, sonication induces the generation of an oil-in-water emulsion. Glycogen molecules then agglomerate at the interface of the oil droplets, encapsulating them. Reproduced from the study published by the authors of [58].

As shown in Table 1, it can be observed that most studies have focused on the encapsulation of polyphenols, which is consistent with it being the most abundant class of phytochemicals. Shell materials, on the other hand, have mostly been polysaccharides, which are biopolymers composed of many subunits linked through α- or β-glycosidic bonds [63]. Figure 5 depicts an example of how these compounds, more specifically glycogen, can act to form the capsule wall in ultrasound-assisted encapsulation processes. Furthermore, different polysaccharides have distinct gastrointestinal behaviors, which can be advantageous to formulate capsules with a target release in the organism [9]. Additionally, these compounds are biocompatible and non-toxic, also being able to act as natural emulsifiers and form gels, which are great qualities for food applications [64].

#### 4.2.4. Incorporation of Phytochemical Capsules into Food Products

Several studies that have been developed in the last five years on the topic of phytochemical encapsulation using US technology have evaluated the potential of incorporating the capsules into food products mainly via the evaluation of bioavailability, stability during storage, and controlled release of the core material [50,51]. However, there is still room for further development in this field by testing the performance of the capsules in model food products. In this way, the effects of real processing conditions (e.g., baking, cooking, or pasteurization processes) on capsule stability and phytochemical recovery could be investigated, as has been the trend with several publications on encapsulation for food processing [65,66,67]. Furthermore, the effects of capsule incorporation into food products on sensory properties should also be evaluated. 

#### 4.2.5. Possibility of Scaling Up

**Figure 6:**
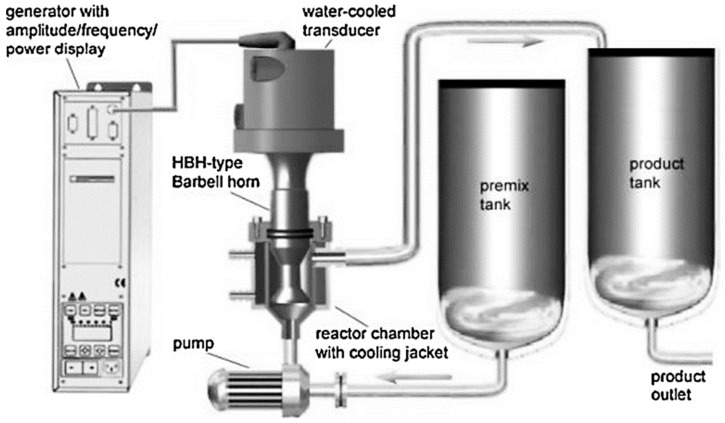
Instrumental setup for the scale-up application of the ultrasound-assisted nanoemulsification process using a single pass flow-through approach. The aqueous and oil phases are mixed in the premix tank, and then pumped through the reactor chamber (where emulsification occurs) into the product tank. HBH: half-wave Barbell horn. Reproduced from the study published by the authors of [70].

One of the major challenges that remain for US applications is the difficulty of process scale-up due to instrumental limitations, which are mainly related to the depth of wave propagation and the scale of the transducers [68]. Specifically on the field of ultrasound-assisted encapsulation, very few applications exist on this subject. No applications were found on this topic for this review article, but several systems have been developed for the scale up of ultrasound-assisted emulsification processes for drug delivery, which have applied probe-type US systems with a flow-through approach (Figure 6) [69,70]. 

In both of these applications, a half-wave Barbell horn (HBH), with a 32 mm tip, was used in a flow-through reaction chamber with a controlled temperature. The HBH was operated at 20 kHz and a 90 µm peak-to-peak amplitude. The temperature varied, with one study using 40 °C [70], and the other reporting that temperature was maintained below 60 °C [69]. In these systems, the aqueous and oil phases were previously mixed, then pumped through the reactor, where the nanoemulsion was formed, and into the product tank. This approach enabled processing up to around 4 kg of sample in 60 min in one of the studies [69], and a productivity of 2.5 L min^−1^ was achieved for the other, which was reported to be eight times faster than the conventional method [70]. In this sense, this type of system can present a promising strategy for application in the US-assisted encapsulation of phytochemicals based on the emulsification technique. However, further studies are necessary to evaluate the suitability of the process parameters and compatibility with other encapsulation mechanisms using US.

## 5. Conclusions

The use of US for the encapsulation of phytochemicals aiming at increasing food functionality has drastically increased over the last decade, and this is still a developing field. Ultrasound has been used as a stand-alone technique for encapsulation via the emulsification technique, but it has also been used together with other techniques (such as coacervation, liposome entrapment, freeze drying, and spray drying) to increase efficiency and achieve longer shelf lives, or to facilitate transportation. The main focus, in the last five years, has been the encapsulation of polyphenols, and most shell materials have been composed of polysaccharides, such as chitosan, starch and zein, although oils and phospholipids have also been applied. 

A lack of standard terminology regarding the use of US was observed in this field, especially regarding the classification of processes as emulsification or encapsulation. Furthermore, very little data were given on the processing parameters regarding sonication. In many studies, essential information is missing, such as the power, frequency, amplitude, or even the reactor type of the US system. In addition, different US parameters were usually not evaluated, with only one condition being applied. In this sense, there is a need to fill this gap in the field of the US-assisted encapsulation of phytochemicals, so that a higher understanding of the underlying US mechanisms in these processes can be achieved. For this to happen, more comprehensive optimizations of the process parameters (including, at least, the evaluation of US frequency, US power, temperature, and time for new systems) should be performed in future studies. Furthermore, studies in which the treatment has been previously optimized should still report the system type, US frequency, output power, temperature, treatment time, and US intensity or density, which are key parameters in the context of US processing.

Overall, there was a tendency for studies to evaluate capsule behavior not only during simulated storage, but also in in vitro digestion assays to assess their bioavailability. Furthermore, several studies have studied the controlled release of the encapsulated phytochemicals, as well as capsule cytotoxicity. However, no studies evaluated the incorporation of the produced capsules into model foods, which can be important to evaluate stability during food processing and assess the suitability of the sensory properties of the product with consumer taste. Hence, there is still potential for more in-depth analyses of the characteristics of the phytochemical capsules produced via US technology by simulating actual processing conditions and evaluating the sensory properties of the final product. Finally, there is a lot of room for the development of scale-up systems using the flow-through approach, which has already been investigated for a number of ultrasound-assisted emulsification processes and has great potential for this field. 

## Figures and Tables

**Table 1:** Applications of ultrasound for phytochemical encapsulation aiming at incorporation in food products.

Core Composition	Shell Composition	Encapsulation Technique	Experimental Conditions	Remarks	Reference
Polyphenols, iridoids, anthocyanins, and tannins (from cornelian cherry)	β-cyclodextrin	Emulsification followed by freeze drying	Type of US reactor: bath US frequency: 37 kHz US power: 550 W US amplitude: n.i. Time: 20 min Temperature: 30 °C Observations: 50 mL of solvent per gram of plant. Solvent consisted of a 1.5% β-cyclodextrin solution in 50% ethanol (w w^−1^)	Extraction and encapsulation of phytochemicals were performed in the same procedure. An encapsulation efficiency of 65.62% was achieved.	[52]
Resveratrol	Zein–chitosan	Coacervation	Type of US reactor: bath US frequency: single- (28 or 40 kHz), dual- (28/40 kHz), or multi- (20/28/40 kHz) frequency mode US power: 300 W US amplitude: n.i. Time: 40 min (pulsed mode: 10 s on/3 s off) Temperature: 25 ± 2 °C (a water bath was used) Observations: Different zein: chitosan ratios were evaluated (from 1:1 to 1:2.5). A sample volume of 3 L was used. The samples were kept away from light during sonication. Capsules were freeze dried after synthesis.	Regarding complex ratios, the 1:2.5 zein: chitosan mixture presented the highest encapsulation efficiency (51.4%). Regarding US frequency, the 20/40 kHz treatment presented the highest encapsulation efficiency (65.2%), which was 32% higher than the control. The US treatment significantly decreased the capsule size and polydispersity index.	[50]
Hydrolyzed collagen (from sea bass skin) conjugated with 3% epigallocatechin gallate	Phospholipids (soy lecithin: cholesterol, 4:1)	Liposome entrapment	Type of US reactor: bath or probe US frequency: n.i. (bath) and 20 ± 0.05 kHz (probe) US power: n.i. (bath) and 750 W (probe) US amplitude: n.i. (bath) and 20 or 40% (probe); Time: 30 min (bath) and 2, 5, 10, or 15 min (probe; pulsed mode: 1 s on/ 10 s off) Temperature: 25 ± 5 °C (an ice water bath was used) Observations: 35 mL of 1% core material was added to the phospholipid film, which was then sonicated using bath or probe US.	Probe US resulted in a lower encapsulation efficiency than bath US, which was more pronounced with longer times and higher amplitude. Probe US resulted in a smaller capsule size, lower polydispersity index, and higher negative surface charge. A higher encapsulation efficiency and antioxidant activity, as well as a lower capsule size after storage (28 days) were also observed. US had no effect on the release rate of core material. Probe US resulted in a higher in vitro bioavailability.	[51]
Phenolic compounds (from mango peel extract)	Sunflower oil (continuous phase of first emulsion) and distilled water (continuous phase of second emulsion)	Double emulsification (water-in-oil-in-water)	Type of US reactor: probe (22 mm sonotrode) US frequency: 24 kHz US power: 400 W US amplitude: 40, 60, 80 or 100 µm (first emulsion); 50 µm (second emulsion) Time: 1, 2, or 3 min (first emulsion); 1.5 min (second emulsion) Temperature: n.i. Observations: first emulsion medium—extract in 0.1 mol L^−1^ NaCl (22%), corn oil (70%), polyglycerol polyricinoleate (5%), and glycerol (3%). Second emulsion medium—first emulsion (25%) and 2% of hydrophilic surfactant in 0.1 mol L^−1^ NaCl (25%). Pre-emulsification was performed via mechanical agitation (6000 rpm for 8 min for the first emulsion and 3000 rpm for 4 min for the second emulsion).	The US treatment using 100 µm of amplitude for 3 min (first emulsion step) resulted in the lowest capsule size (0.38 µm). The highest encapsulation efficiency was obtained using Tween 20 as the surfactant for the second emulsion (98.65 ± 1.14%). The highest encapsulation stability after storage (21 days) was obtained using Tween 80 as the surfactant for the second emulsion.	[31]
Phenolic compounds	Chitosan	Simple coacervation followed by freeze drying	Type of US reactor: bath US frequency: 42 kHz US power: n.i. US amplitude: 70% Time: 4.79 min Temperature: room temperature Observations: The optimum conditions for nanoencapsulation were a mixture of 0.28% chitosan and 0.29% sodium tripolyphosphate in the ratio of 5:1 and pH of 4.9. The obtained capsules were freeze dried.	A power supply of 230 V was used. The nanoencapsulation efficiency was 69.9 ± 0.67%, with a particle size between 72.3 and 460.7 nm, along with a polydispersity index of 0.458. A ζ potential of +15.73 mV was obtained.	[53]
Quercetin	Oleic acid and sodium caseinate	Emulsification	Type of US reactor: probe US frequency: n.i. US power: 300 W US amplitude: n.i. Time: 5 min Temperature: room temperature Observations: A 5 mg mL^−1^ quercetin solution in ethanol was used. Oleic acid and sodium caseinate mixtures were evaluated in the ratios of 1:40, 1:15, 1:8, or 1:4 (m m^−1^). After sonication, the mixture was kept in the dark for 30 min.	A polydispersity index below 0.3 and a ζ potential higher than 30 mV were obtained. The bioaccessibility of quercetin increased from 25% to more than 60% after encapsulation.	[54]
Rutin	Quinoa and maize starch	Emulsification followed by freeze drying	Type of US reactor: probe US frequency: 20 kHz US power: n.i. US amplitude: n.i. Time: 30 min (in 3 min intervals) Temperature: 80 °C Observations: A solution of 1.5% rutin in ethanol was dripped into 1.5% starch suspensions containing 0.1 mol L^−1^ NaOH at 80 °C (in the ratio of 1:10) over 30 min with continuous stirring prior to sonication.	Capsule sizes between 107 and 222 nm, encapsulation efficiencies of 67.4 and 63.1%, and ζ potentials of −18.0 and −18.6 mv, were obtained for the quinoa and maize starch capsules, respectively. Rutin’s bioavailability was significantly higher in quinoa capsules.	[55]
Ferulic acid	Modified quinoa and maize starch	Sonoprecipitation followed by freeze drying	Type of US reactor: probe US frequency: 20 kHz US power: 750 W US amplitude: 80% Time: 10 min Temperature: 25 (an ice water bath was used) Observations: 1.5% starch suspensions in 0.1 mol L^−1^ NaOH were preheated at 80 °C and stirred for 30 min prior to sonication. Afterwards, starch nanoparticles were precipitated through the addition of ethanol in the ratio of 1:2, centrifuged, and freeze dried.	Particle sizes were 153 and 221 nm for quinoa and maize starch capsules, respectively. The polydispersity index was lower than 0.4. Zeta potentials for quinoa and maize starch were 24.2 and 16.2 mV, respectively. Quinoa-based capsules presented a higher encapsulation efficiency.	[56]
Phytosterols	Flavor oil	Emulsification followed by spray drying	Type of US reactor: probe US frequency: 20 kHz US power: 200, 280, and 360 W US amplitude: n.i. Time: 5 min (pulsed mode: 5 s on/ 5 s off) Temperature: n.i. (an ice water bath was used) Observations: the reaction medium consisted of flavor oil (20%), tea saponin (3%), phytosterols (4%), and aqueous buffer (pH 7). Emulsions were spray dried.	The higher treatment time led to a smaller capsule size.	[27]
Anthraquinones (aloin, aloe-emodin, and rhein) extracted from aloe vera	Casein micelles	Emulsification	Type of US reactor: probe US frequency: 20 kHz US power: 500 W (input power); 39.74 W (output power) US amplitude: 50% US intensity/density: n.i. Time: 15 min (pulsed mode: 30 s on/ 30 s off) Temperature: 25 °C (ice tube) Observations: no observations.	The particle size and ζ potential of capsules ranged from 171 to 179 nm and −23 to −17 mV, respectively. Encapsulation efficiencies ranged from 98 to 100%.	[57]
Soybean oil and vitamin D	Glycogens (from rabbit and bovine liver, oyster, or sweet corn)	Emulsification	Type of US reactor: probe US frequency: 20 kHz US power: 160 W US amplitude: n.i. Time: 45 s Temperature: room temperature Observations: the medium consisted of 10 mg mL^−1^ of different glycogens and 50 µL of the oil phase. The capsules were isolated via flotation and washing with ultrapure water.	Microcapsules with sizes varying from 0.3 to 8 µm were obtained.	[58]
Sacha inchi oil	Alginate and chitosan	Emulsification followed by coacervation	Type of US reactor: probe US frequency: n.i. US power: n.i. US amplitude: n.i. Time: 3 min Temperature: 7 to 10 °C Observations: Samples containing a surfactant, the core, and the shell materials were sonicated. Then, coacervation was performed using alginate, followed by coating with chitosan.	The obtained nanoparticles presented a higher loading efficiency and stability in comparison to other vegetable oils.	[59]

n.i.—not informed.

## Data Availability

No new data were created or analyzed in this study. Data sharing is not applicable to this article.
